# Supplementary material for: SARS-CoV-2 Omicron Induces Enhanced Mucosal Interferon Response Compared to other Variants of Concern, Associated with Restricted Replication in Human Lung Tissues
Source: Viruses. 2022 Jul 21;14(7):1583. doi: 10.3390/v14071583 (PMC9318963; doi:10.3390/v14071583)
Supplement: Supplementary file 1 [file viruses-14-01583-s001.zip › viruses-1815015-Supplementary.pdf]

Supplementary Material

Supplementary Figures

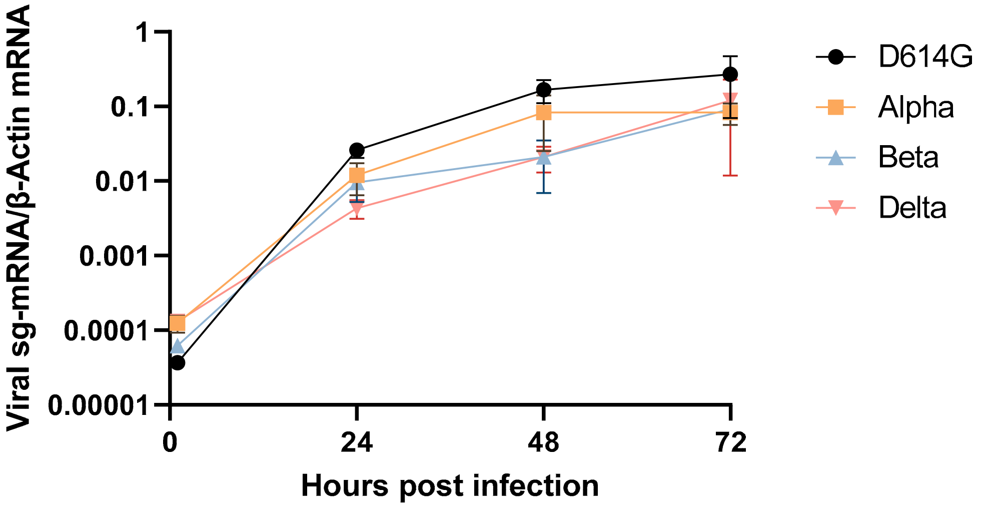

Figure S1. Replication kinetics of SARS-CoV-2 variants in human lung tissues. Lung organ cultures were infected in parallel with the indicated variants. Levels of tissue-associated SARS-CoV-2 N gene subgenomic (sg)-mRNA were determined by RT-qPCR and normalized to β-actin. The data shown represent the mean values (± SEM) of at least three independent tissues, each tested in 4 biological replicates.

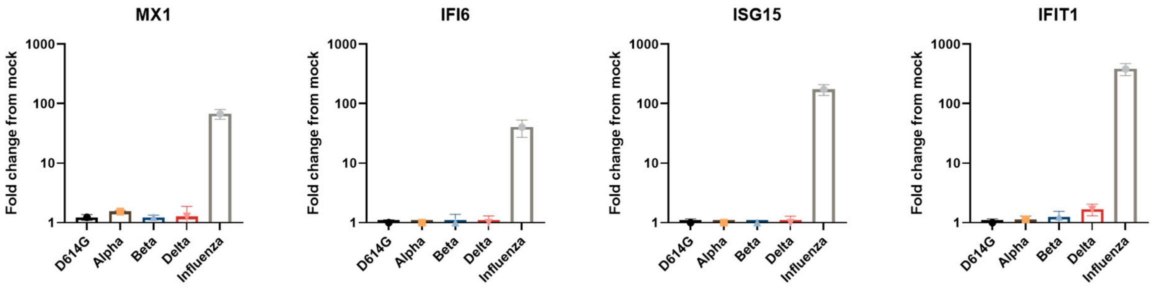

Figure S2. Lung tissue ISG response to SARS-CoV-2 variants and influenza. Lung organ cultures were infected in parallel with the indicated variants, and with Influenza A/H1N1 (10^5 PFU/well). RNA was extracted from mock- and infected tissues at 24h post infection, and the effect of infection by the indicated viruses on the expression of interferon-stimulated genes (ISG) is presented as fold-change from mock infection. The data shown represent the mean values (± SEM) of at least three independent tissues, each tested in 4 biological replicates.

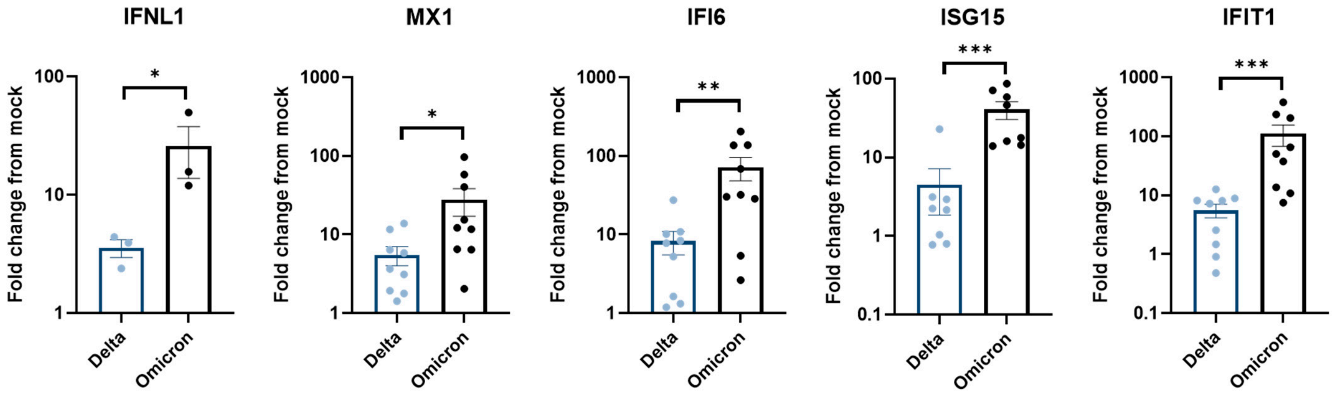

Figure S3. Nasal tissue ISG response to SARS-CoV-2 Omicron and Delta. Nasal organ cultures were infected in parallel with Omicron and Delta (10^5 PFU/well), and the effect of infection on the expression of the indicated interferon-stimulated genes (ISG), measured by RT-qPCR at 24h post infection, is presented as fold-change from mock-infection. The data shown represent the mean values (± SEM) of 3 independent tissues (for IFNL1) and 9 independent tissues (for MX1, IFI6, ISG15, and IFIT1), each tested in 4 biological replicates. *, $P < 0.05$; **, $P < 0.01$; ***, $P < 0.001$

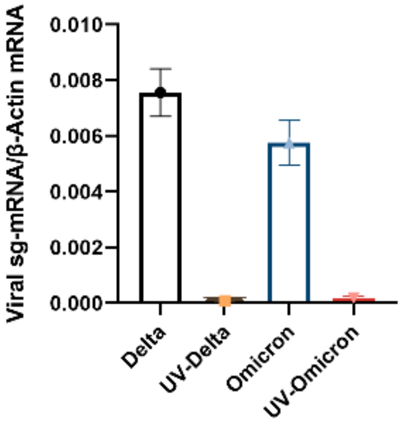

Figure S4. Viral subgenomic (sg)-mRNA in lung tissues exposed to infectious versus UV-inactivated SARS-CoV-2 Omicron and Delta. Lung organ cultures were exposed in parallel to infectious versus UV-inactivated Omicron and Delta variants (10^5 PFU/well), and the levels of tissue-associated viral sg-mRNA were measured by RT-qPCR at 24h post infection. The results shown as mean values (± SEM) in a representative tissue (tested in 4 biological replicates), represent at least three independent lung tissues.

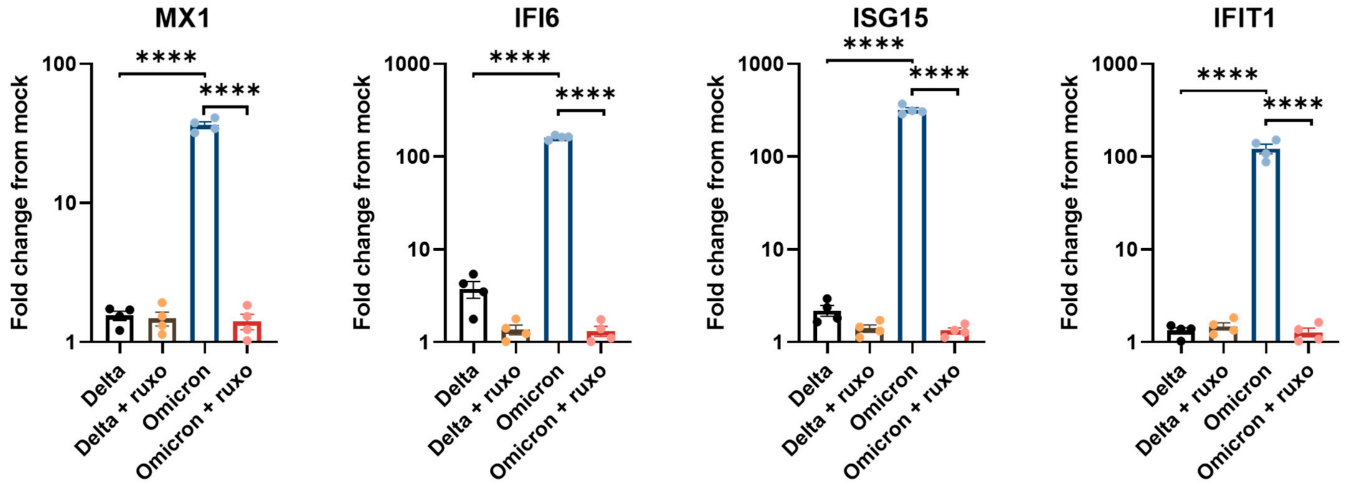

Figure S5. Effect of Ruxolitinib on lung tissue ISG response to Omicron infection. Lung organ cultures were pretreated with 5uM Ruxolitinib (Ruxo) for 16 h when indicated and infected in parallel with Omicron and Delta (10^5 PFU/well). The effect of infection with or without Ruxo on the expression of the indicated interferon-stimulated genes (ISG), measured by RT-qPCR at 24h post infection, is presented as fold-change from mock-infection. The data shown represent the mean values (± SEM) of 4 biological replicates. ***, $P < 0.001$; ****, $P < 0.0001$
